# A radiomics-based model on non-contrast CT for predicting cirrhosis: make the most of image data

**DOI:** 10.1186/s40364-020-00219-y

**Published:** 2020-09-17

**Authors:** Jin-Cheng Wang, Rao Fu, Xue-Wen Tao, Ying-Fan Mao, Fei Wang, Ze-Chuan Zhang, Wei-Wei Yu, Jun Chen, Jian He, Bei-Cheng Sun

**Affiliations:** 1 Department of Hepatobiliary Surgery of Drum Tower Clinical Medical College, Nanjing Medical University, Nanjing, China; 2 Department of Hepatobiliary Surgery, The Affiliated Drum Tower Hospital of Nanjing University Medical School, 321 Zhongshan Road, Nanjing, 210008 Jiangsu Province China; 3 Department of Radiology, The Affiliated Drum Tower Hospital of Nanjing University Medical School, 321 Zhongshan Road, Nanjing, 210008 Jiangsu Province China; 4 Department of Pathology, The Affiliated Drum Tower Hospital of Nanjing University Medical School, 321 Zhongshan Road, Nanjing, 210008 Jiangsu Province China

**Keywords:** Hepatitis B virus (HBV), Liver cirrhosis, Non-contrast computed tomography (CT), Radiomics model

## Abstract

**Background:**

To establish and validate a radiomics-based model for predicting liver cirrhosis in patients with hepatitis B virus (HBV) by using non-contrast computed tomography (CT).

**Methods:**

This retrospective study developed a radiomics-based model in a training cohort of 144 HBV-infected patients. Radiomic features were extracted from abdominal non-contrast CT scans. Features selection was performed with the least absolute shrinkage and operator (LASSO) method based on highly reproducible features. Support vector machine (SVM) was adopted to build a radiomics signature. Multivariate logistic regression analysis was used to establish a radiomics-based nomogram that integrated radiomics signature and other independent clinical predictors. Performance of models was evaluated through discrimination ability, calibration and clinical benefits. An internal validation was conducted in 150 consecutive patients.

**Results:**

The radiomics signature comprised 25 cirrhosis-related features and showed significant differences between cirrhosis and non-cirrhosis cohorts (*P* < 0.001). A radiomics-based nomogram that integrates radiomics signature, alanine transaminase, aspartate aminotransferase, globulin and international normalized ratio showed great calibration and discrimination ability in the training cohort (area under the curve [AUC]: 0.915) and the validation cohort (AUC: 0.872). Decision curve analysis confirmed the most clinical benefits can be provided by the nomogram compared with other methods.

**Conclusions:**

Our developed radiomics-based nomogram can successfully diagnose the status of cirrhosis in HBV-infected patients, that may help clinical decision-making.

## Introduction

As reported by the World Health Organization (WHO), chronic hepatitis B (CHB) has been a major public health problem with an estimated 240 million infectors and 650,000 deaths due to it [1]. The complications of CHB mainly include cirrhosis and hepatocellular carcinoma, leading to poor prognosis [2]. CHB is highly endemic in China with more than 74 million hepatitis B virus (HBV) surface antigen (HBsAg) carriers [3, 4]. For controlling the spread of CHB in China, it is necessary to conduct early diagnosis and therapeutic interventions in allusion to HBV.

Liver cirrhosis is a diffuse hepatic process characterized by fibrosis and structurally abnormal nodules, with serious complications (e.g. gastroesophageal varices, ascites, hepatic encephalopathy, and renal and cardiac disturbances), which has been reported as a serious cause of death in all developed countries [5]. According to 2017 European Association for the Study of Liver (EASL) guideline for HBV [6], it was emphasized that HBV-infected patients with compensated or decompensated cirrhosis require treatment regardless of HBV DNA or alanine aminotransferase (ALT) levels. Therefore, early detection of cirrhosis is needed. Liver biopsy is traditionally regarded as a reference standard for staging fibrosis [7]. However, it was restricted by several defects including invasiveness, expensiveness and sample biases [8–11]. For this, various noninvasive tests have been developed in the past decades [12]. Noninvasive staging mainly depends on serum biomarkers-based formula or elasticity-based imaging techniques (such as transient elastography [TE], also called Fibroscan). In China, Fibroscan is not widespread because of its high cost (€34,000 for a portable device and €5000 for its annual maintenance). Liver stiffness of patients cannot be measured during physical examination. Hepatocellular carcinoma often occurs in patients with chronic hepatitis or cirrhosis [13], and 2018 practice guidance of the American Association for the Study of Liver Diseases (AASLD) did not recommend contrast-enhanced computed tomography (CT) and magnetic resonance image (MRI) for tumor surveillance due to limited cost-effectiveness [14]. Although CT is frequently suggested for HBV carriers in annual clinical examinations in China, many patients only accepted non-contrast CT examinations.

Radiomics is a new field of image analysis technology, which can covert images into large amount of quantitative data for more biological information [15]. Several studies have used radiomics on shear wave elastography or MRI for the prediction of liver fibrosis [16, 17]. Considering non-contrast CT is easily obtained in clinical examinations, the predictive value is worth detecting with radiomics. The aim of this study was to establish a radiomics-based model on non-contrast CT for the prediction of liver cirrhosis.

## Materials and methods

This retrospective study was approved by the institutional review board of our institution. The requirement for written informed consent was waived due to its retrospective nature.

### Patients

**Fig. 1:**
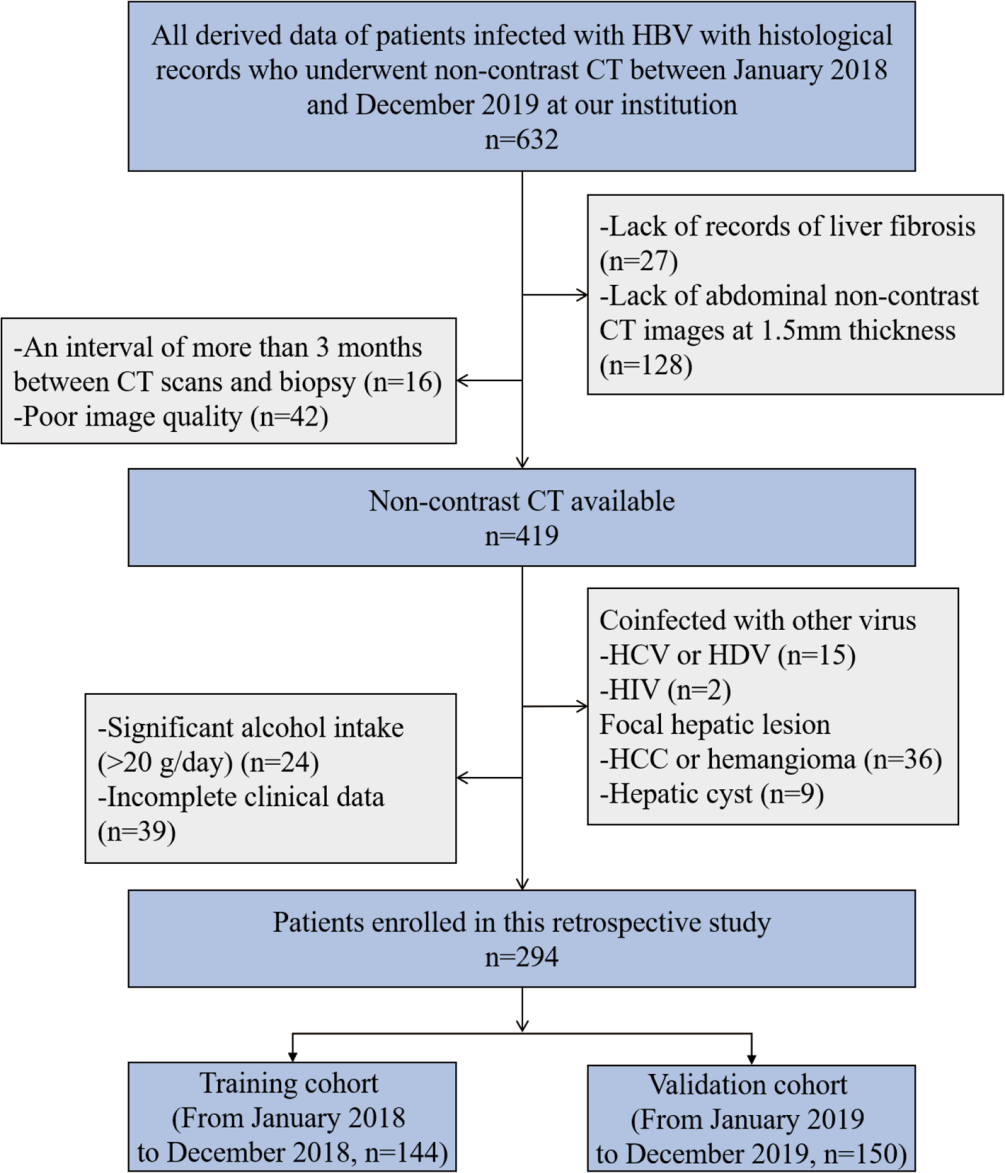
Patient selection flow chart. HBV, hepatitis B virus; HCC, hepatocellular carcinoma; HCV, hepatitis C virus; HDV, hepatitis D virus; HIV, human immunodeficiency virus

632 HBV-infected patients with pathologic results of liver fibrosis who underwent non-contrast CT at our institution from January 2018 to December 2019 were retrospectively reviewed. The exclusion criteria were: (1) lack of pathological records of liver fibrosis (*n* = 27); (2) lack of abdominal non-contrast CT images at 1.5 mm thickness (*n* = 128); (3) an interval of more than 3 months between CT scans and biopsy (*n* = 16); (4) poor image quality (*n* = 42); (5) co-infected with other virus (e.g. hepatitis C virus [HCV], hepatitis D virus [HDV] and human immunodeficiency virus [HIV]) (*n* = 17); (6) focal hepatic lesion (e.g. hepatocellular carcinoma, hepatic tuberculosis and any other) (*n* = 45); (7) significant alcohol intake (> 20 g/day) (*n* = 24); (8) incomplete clinical data (*n* = 39). 294 patients were finally included in this study (Fig. 1). Based on the date of biopsy, 144 patients between January 2018 and December 2018 were allocated to the training cohort, and 150 between January 2019 and December 2019 were for validation. The dates of clinical data were at the first diagnosis of CHB. Table 1 shows their baseline characteristics.

**Table 1:** Baseline characteristics

Parameter	Training (***n*** = 144)	Validation (***n*** = 150)	***P*** value
**Sex**			.88
**No. of men**	117 (81.3)	120 (80.0)	
**No. of women**	27 (18.7)	30 (20.0)	
**Age**			.49
**< 60 (years)**	68 (47.2)	77 (51.3)	
**≥ 60 (years)**	76 (52.8)	73 (48.7)	
**CT-reported cirrhosis**			.81
**Cirrhosis-negative**	98 (68.1)	100 (66.7)	
**Cirrhosis-positive**	46 (31.9)	50 (33.3)	
**Laboratory findings**^a^			
**AST (IU/mL)**	31.6 (23.5–48.2)	29.3 (21.1–38.5)	.13
**ALT (IU/mL)**	31.2 (22.0–50.0)	26.9 (19.6–39.6)	.07
**GGT (IU/mL)**	53.5 (32.0–111.4)	54.9 (29.5–91.2)	.26
**Total bilirubin (ng/mL)**	13.5 (9.2–18.5)	12.9 (10.0–17.8)	.16
**Platelet count (10**^**9**^**/L)**	141.5 (91.8–182.3)	138.5 (100.5–188.3)	.70
**INR**	1.04 (0.98–1.11)	1.04 (0.98–1.11)	.31
**APRI**	0.63 (0.41–0.98)	0.53 (0.33–0.91)	.16
**FIB-4**	2.65 (1.70–4.14)	2.30 (1.62–3.76)	.21
**Histologic grade**			.59
**F0**	18 (12.5)	14 (9.3)	.46
**F1**	24 (16.7)	32 (21.3)	.37
**F2**	13 (9.0)	18 (12.0)	.45
**F3**	26 (18.1)	29 (19.3)	.88
**F4**	63 (43.8)	57 (38.0)	.34

Note. —Except where indicated, data are numbers of patients, with percentages in parentheses. *ALT* alanine aminotransferase, *APRI* aspartate aminotransferase-to-platelet ratio, *AST* aspartate aminotransferase, *FIB-4* fibrosis-4 index, *GGT* γ-glutamyl transferase, *INR* international normalized ratio

^a^Data are medians, with interquartile range in parentheses

Clinical characteristics and the data of CT scan were obtained from medical records. Clinical data included age, sex, blood routine tests (red blood cell [RBC], white blood cell [WBC], platelet [PLT] count and hemoglobin [Hb]), liver function examinations (ALT, aspartate aminotransferase [AST], alkaline phosphatase [ALP], glutamyl transpeptidase [GGT], lactate dehydrogenase [LDH], total bilirubin [TB], conjugated bilirubin [CB], albumin [ALB], globulin [GLOB], total bile acid [TBA] and leucine arylamidase [LAP]), lipid metabolism tests (total cholesterol [TC], high density lipoprotein cholesterol [HDL-C], low density lipoprotein cholesterol [LDL-C], apolipoprotein A1 [Apo A1] and apolipoprotein B [Apo B]), C reactive protein (CRP) and blood coagulation function (prothrombin time [PT] and international normalized ratio [INR]).

### Liver biopsy

Percutaneous liver biopsy was performed in the right lobe of a liver under the ultrasonic guidance by ultrasonologists. Liver samples were histologically analyzed by two pathologists in consensus. Each of pathologists had more than 5 years of work experience, and they were both blinded to the clinical information. Liver fibrosis was determined according to the Metavir scoring system [18]. F ≥ 2 was considered as significant fibrosis, F ≥ 3 as serious fibrosis and F4 as cirrhosis.

### CT image acquisition and radiologic evaluation

All patients underwent CT examination in the supine position on an identical model CT scanner (Lightspeed, VCT, or Discovery HD 750, GE Healthcare, US). The unified parameters of CT scan were: tube voltage 120 kVp, tube current 250–350 mA, collimating slice thickness 5 mm, reconstruction slice thickness 1.25 mm, slice interval 5 mm, rotation time 0.6 s, helical pitch 1.375, the field of view between 35 and 40 cm, matrix 512 × 512. A standard reconstruction algorithm was applied.

Two radiologists reviewed all non-contrast CT scans to evaluate the presence of cirrhosis for each patient. Image findings suggestive of cirrhosis on CT scans include a nodular or irregular hepatic surface, a blunt liver edge, parenchymal abnormalities, intrahepatic morphological changes and manifestations of portal hypertension [19]. Both radiologists were aware of the diagnosis of CHB but were blinded to the clinical-radiological details. Any disagreement was resolved through consultation.

### Serum fibrosis tests

Because of easily obtained parameters, the aspartate aminotransferase-to-platelet ratio index (APRI) and the fibrosis-4 index (FIB-4) are frequently used for the prediction of fibrosis. Formulas are shown as below [20, 21]:

$$ \mathrm{APRI}=\frac{\left(\mathrm{AST}\left(\mathrm{IU}/\mathrm{L}\right)/\mathrm{ULN}\right)\times 100\ }{\mathrm{Platelet}\ \mathrm{count}\ \left({10}^9/\mathrm{L}\right)} $$

$$ \mathrm{FIB}-4=\frac{\mathrm{age}\left(\mathrm{years}\right)\times \mathrm{AST}\left(\mathrm{IU}/\mathrm{L}\right)\ }{\mathrm{Platelet}\ \mathrm{count}\ \left({10}^9/\mathrm{L}\right)\times \mathrm{ALT}\left(\mathrm{IU}/\mathrm{L}\right)\hat{\mkern6mu} 1/2} $$

These two indices were calculated using results of laboratory tests within a month from biopsy.

### Radiomic feature extraction and selection

**Fig. 2:**
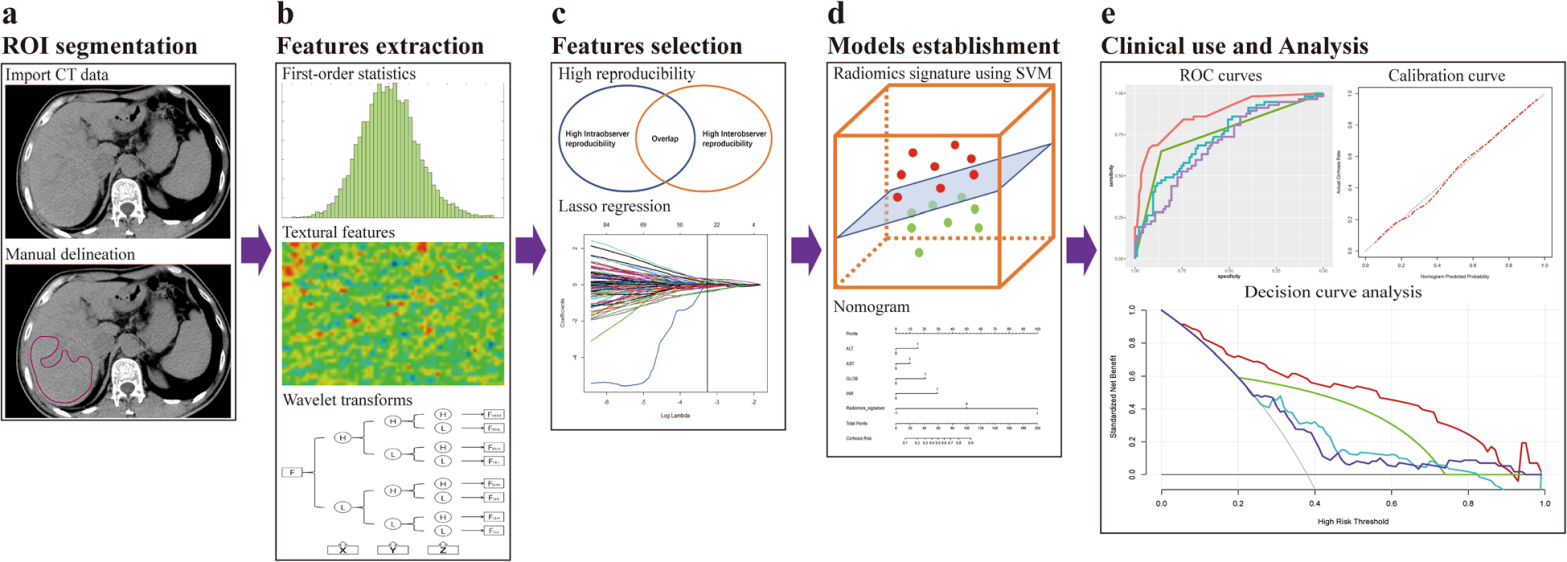
Workflow of necessary steps in this study. **a** ROI was manually delineated on non-contrast CT scans at the level of right portal veins. **b** Radiomic features including first-order statistics, textural features and wavelet transforms were extracted. **c** Intra- and interobserver reproducibility and subsequent lasso regression were used for feature selection. **d** A radiomics signature was constructed with SVM and a radiomics-based nomogram integrates radiomics signature and clinical predictors. **e** The performance of established models was evaluated by ROC, calibration and DCA curves. ROI, region of interest; LASSO, least absolute shrinkage and selection operator; SVM, support vector machine; ROC, receiver operator characteristic; DCA, decision curve analysis

The workflow is depicted in Fig. 2. Two radiologists (reader 1&2) were involved in image segmentation and radiomic feature extraction. Reader 1 selected region of interest (ROIs) in the liver of all patients using 3D slicer (version 4.8.0; http://www.slicer.org). ROIs for the liver were delineated along the margin of the right hepatic lobe, at the level of the right portal vein, by excluding large hepatic vessels on non-contrast CT images (mean area of ROIs, 47 cm^2^ ± 15; range, 19–106 cm^2^) (Fig. S1). Image preprocessing and feature extraction were performed with the open-source Pyradiomics package (http://www.radiomics.io/pyradiomics.html). The voxel spacing was standardized with the size of 1 × 1 × 1 mm and voxel intensity values were discretized with a bin width of 25 HU to reduce the interference of image noise and normalize intensities [22]. Eight hundred twenty-eight radiomic features (18 first-order statistics, 74 textural features, and 736 wavelet-based transformations) were extracted from each ROI. Values of features were standardized using z-scores in the training cohort; z-scores which was applied in the validation cohort used the mean and standard deviation determined in the training cohort.

The reproducibility of each radiomic feature was quantified using intra- and interobserver intraclass correlation coefficient (ICC) based on 50 randomly chosen patients. Reader 1 repeated ROI segmentation twice in a week and reader 2 independently performed ROI segmentation to calculate intra- and interobserver reproducibility, respectively. The minimum acceptable threshold of ICC in this study was 0.8 [23].

**Fig. 3:**
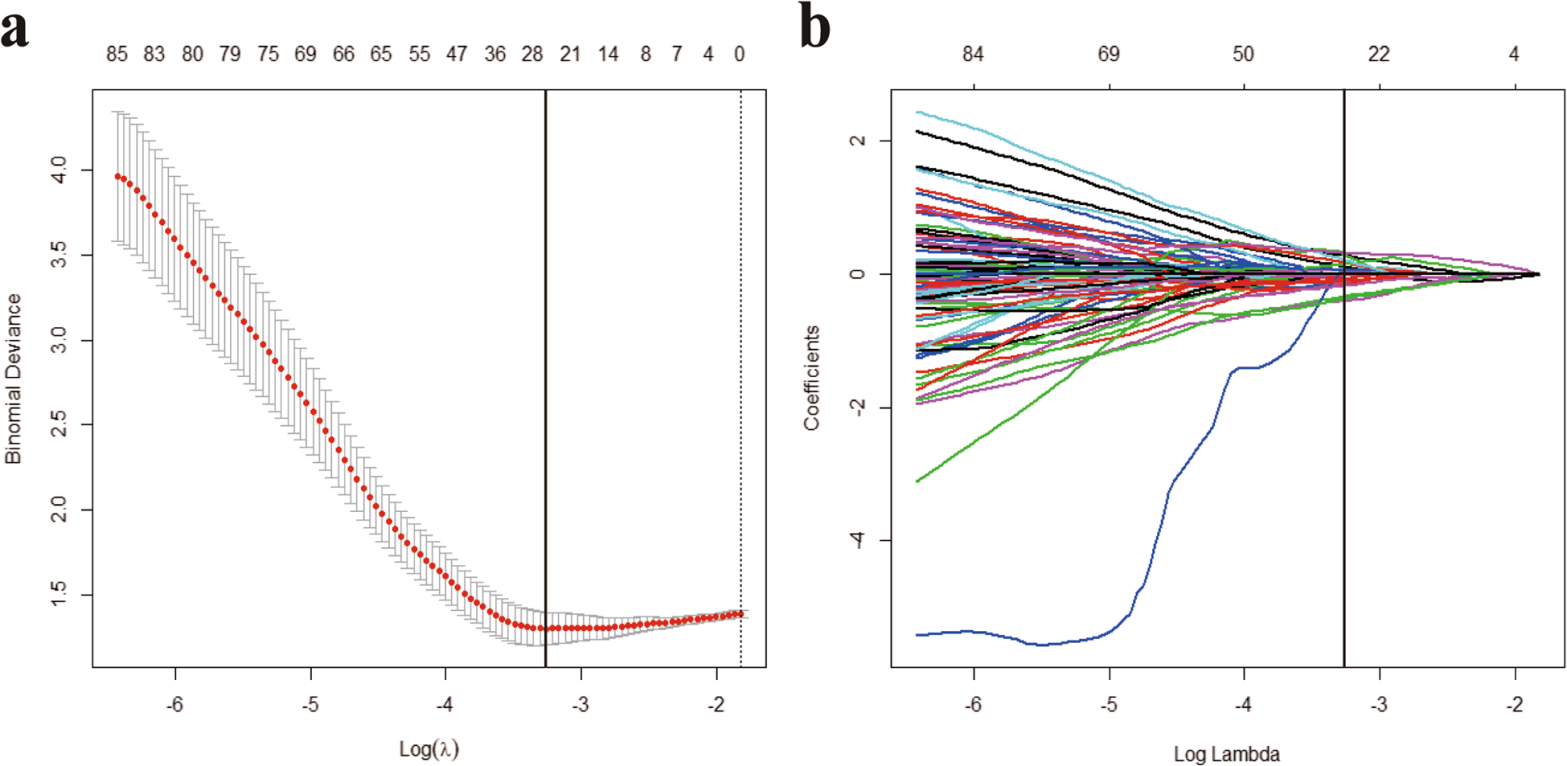
Selections of radiomic features using the least absolute shrinkage and selection operator (LASSO) regression. **a** Optimal λ value was determined by the LASSO model using 10-fold cross-validation via minimum criteria. The binomial deviance curves were plotted versus log(λ). Dotted vertical lines were drawn at the optimal values by using the minimum criteria and the 1 standard error of the minimum criteria (the 1 – standard error criteria). The optimal λ value of 0.0383 was chosen. **b** LASSO coefficient profiles of the 85 selected features is presented

A two-step procedure was followed to select significant radiomic features. First, features with high reproducibility (ICC > 0.8 in both intra- and interobserver settings) were kept for subsequent analysis. Second, the least absolute shrinkage and selection operator (LASSO) logistic regression algorithm [24], with penalty parameter tuning conducted by 10-fold cross-validation, was used to select cirrhosis-related features (with nonzero coefficients) (Fig. 3).

### Radiomics model establishment in the training cohort

The radiomics model for predicting cirrhosis (R-cirrhosis) was established as a binary classifier to distinguish between stages F0–3 and F4. Support vector machine (SVM) was performed based on selected radiomic features for training model by using “e1071” package (https://CRAN.R-project.org/package=e1071) on R software (version 3.6.1, http://www.r-project.org).

### Clinical factors selection

We devised a two-step procedure for selection of clinical factors. First, we used spearman correlation analysis to preliminarily screen out factors with significant correlation (spearman correlation analysis, *P* < 0.05) for subsequent analysis. Second, forward conditional logistic multivariable analysis (input and output *P* value: 0.05 and 0.1, respectively) was used to select factors for predicting cirrhosis. The details are described in Supplement Materials and Methods. The cutoff value of each independent factor was determined by receiver operating characteristic (ROC) analysis (maximizing the Youden index).

### Development and validation of a radiomics-based model

Multivariate logistic regression analysis was performed to establish a model for predicting cirrhosis in the training cohort. A Nomogram was constructed to provide a more understandable outcome measure. The performance of models was subsequently internally tested in the independent validation cohort by using the formula and cutoff values derived from the training cohort. Details are described in Supplement Materials and Methods.

### Statistical analysis

Categorical and continuous variables were compared with χ^2^ test and the Mann-Whitney *U* test, respectively. All statistical analyses were performed using R software (version 3.6.1, http://www.r-project.org). The diagnostic performance of established models was evaluated by the ROC curve and area under the curve (AUC) value. Delong test was used to compare AUC values. Decision curve analysis (DCA) was used to calculate the net benefit from the use of models at different threshold probabilities. Calibration curves were plotted to assess the calibration of the radiomics model, accompanied by the Hosmer-Lemeshow test. A two-sided *P* value less than .05 was considered statistically significant.

## Results

### Baseline characteristics

As summarized in Table 1, there were no differences in clinical-radiological-pathological characteristics between the training and validation cohorts. No differences were found in rates of cirrhosis between the two cohorts (Training: 43.8%, 63 of 144; Validation: 38.0%, 57 of 150; *P* = .34). The overall discrimination accuracy of the CT report of cirrhosis was 77.6% (228 of 294), with a sensitivity of 62.5% (75 of 120), a specificity of 87.9% (153 of 174), positive predictive value of 78.1% (75 of 96) and negative predictive value of 77.3% (153 of 198).

### Radiomics analysis

Of 828 extracted features, 85 features (8 first-order statistics, 21 textural features, and 56 wavelet-based transformations) with high reproducibility were selected for subsequent analysis. Twenty-five cirrhosis-related features with nonzero coefficients in the lasso regression model were selected based on the training cohort (Fig. 3).

A radiomics signature was constructed using SVM algorithm (Supplement Materials and Methods). A difference in radiomics score was obtained between patients with and those without cirrhosis in the training cohort (mean, 0.279 vs − 0.649, *P* < .001), and then confirmed in the validation cohort (mean, 0.141 vs − 0.585, *P* < .001). The radiomics signature showed favorable discriminatory ability with an AUC of 0.879 (95% confidence interval [CI]: 0.827, 0.932) in the training cohort and 0.858 (95% CI: 0.795, 0.921) in the validation cohort.

### Cirrhosis-related clinical factors

In the training cohort, WBC, PLT, ALT, AST, CB, ALB, GLOB, TBA, TC, LDL-C, Apo B, PT and INR were simultaneously related to cirrhosis (*P* < .05 for all, Spearman correlation analysis). The multivariable conditional logistic regression analysis identified ALT, AST, GLOB and INR as independent cirrhosis predictors (Table 2). Cutoff values of them were 25.9 U/L, 32.6 U/L, 33.9 g/L and 1.10, respectively.

**Table 2:** Clinical characteristics of the training cohort related to cirrhosis

	Spearman correlation analysis		Multivariable analysis		ROC analysis	
Variables	***r***^***2***^ value	***P*** value	***b*** coefficient	***P*** value	AUC	Cutoff value
**Age (years)**	0.021	.08	NA	NA	NA	NA
**Sex (male, female)**	0.003	.50	NA	NA	NA	NA
**RBC (10**^**9**^**/L)**	0.020	.08	NA	NA	NA	NA
**WBC (10**^**9**^**/L)**	0.078	.02	NA	.24	NA	NA
**PLT (10**^**9**^**/L)**	0.106	< .001	NA	.07	NA	NA
**Hb (g/L)**	0.020	.08	NA	NA	NA	NA
**ALT (U/L)**	0.066	.002	0.060	.02	0.65 (0.56, 0.74)	25.9
**AST (U/L)**	0.073	.001	−0.092	.01	0.66 (0.57, 0.75)	32.6
**ALP (U/L)**	0.016	.12	NA	NA	NA	NA
**GGT (U/L)**	0.002	.57	NA	NA	NA	NA
**LDH (U/L)**	0.013	.16	NA	NA	NA	NA
**TB (umol/L)**	0.001	.65	NA	NA	NA	NA
**CB (umol/L)**	0.027	.04	NA	.56	NA	NA
**ALB (g/L)**	0.098	< .001	NA	.51	NA	NA
**GLOB (g/L)**	0.049	.006	0.219	.01	0.63 (0.54, 0.73)	33.9
**TBA (umol/L)**	0.066	.001	NA	.53	NA	NA
**LAP (U/L)**	0.000	.81	NA	NA	NA	NA
**TC (mmol/L)**	0.066	.002	NA	.50	NA	NA
**HDL-C (mmol/L)**	0.000	.86	NA	NA	NA	NA
**LDL-C (mmol/L)**	0.053	.005	NA	.44	NA	NA
**Apo A1 (g/L)**	0.009	.25	NA	NA	NA	NA
**Apo B (g/L)**	0.057	.003	NA	.24	NA	NA
**CRP (mg/L)**	0.006	.34	NA	.19	NA	NA
**PT (s)**	0.142	< .001	NA	.58	NA	NA
**INR**	0.149	< .001	16.558	< .001	0.73 (0.65, 0.82)	1.10

Note. ——*b* coefficients are from multivariable logistic regression. Clinical variables found to be significantly related to cirrhosis through spearman correlation analysis entered into forward conditional logistic multivariate analysis. *ALB* albumin, *ALP* alkaline phosphatase, *ALT* alanine aminotransferase, *Apo A1* apolipoprotein A1, *Apo B* apolipoprotein B, *AST* aspartate aminotransferase, *AUC* area under the curve, *CB* conjugated bilirubin, *CRP* C reactive protein, *GGT* glutamyl transpeptidase, *GLOB* globulin, *Hb* hemoglobin, *HDL-C* high density lipoprotein cholesterol, *INR* international normalized ratio, *LAP* leucine arylamidase, *LDH* lactate dehydrogenase, *LDL-C* low density lipoprotein cholesterol, *PLT* blood platelet, *PT* prothrombin time, *RBC* red blood cell, *ROC* receiver operating characteristic, *TB* serum total bilirubin, *TBA* total bile acid, *TC* total cholesterol, *WBC* white blood cell

### Development, performance, and validation of the established model

**Fig. 4:**
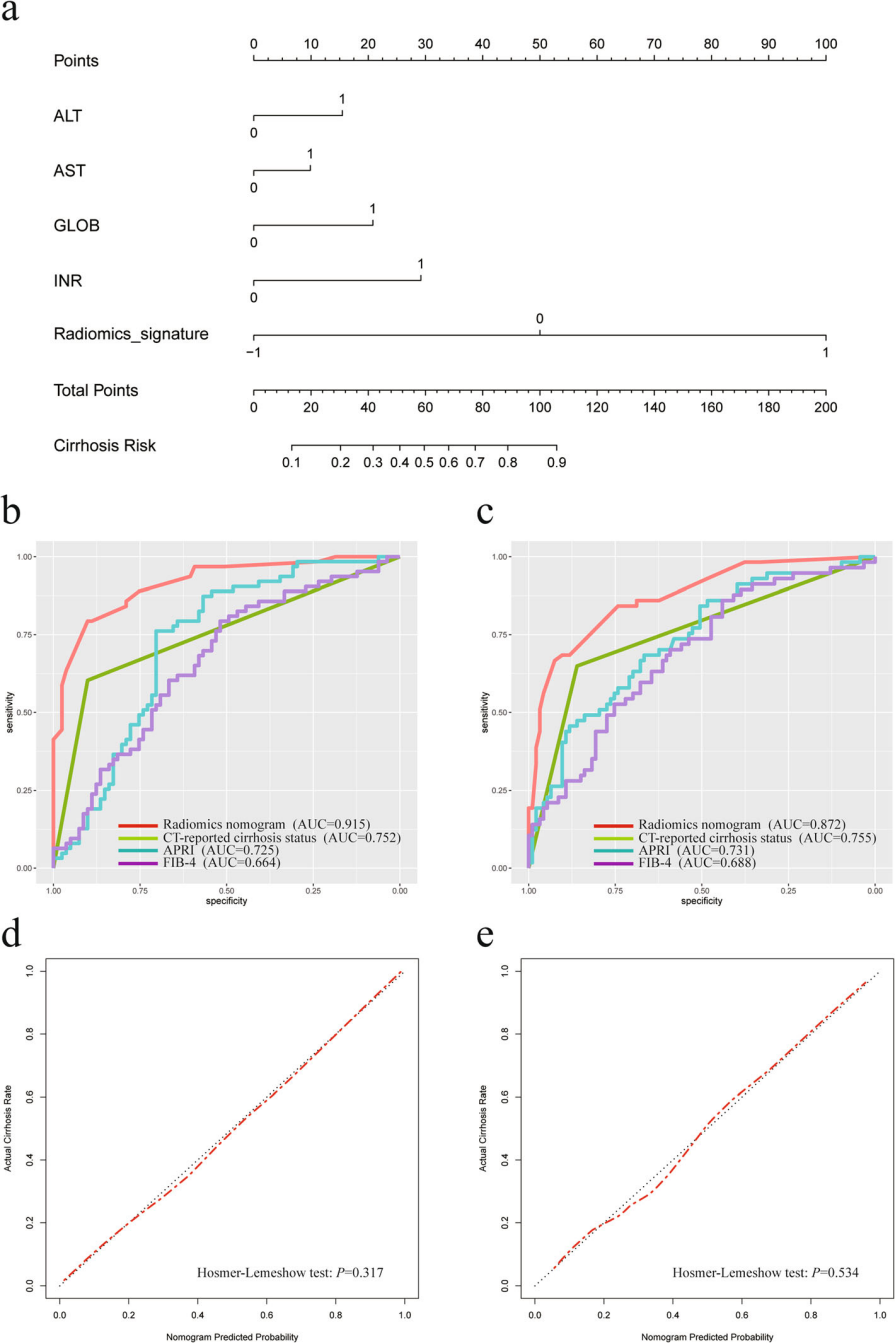
Radiomics nomogram presented with ROC and calibration curves. A radiomics-based nomogram was established due to the training cohort, with radiomics signature, ALT, AST, GLOB and INR incorporated (**a**). Comparison of ROC curves between radiomics nomogram, CT-reported cirrhosis status, APRI and FIB-4 in the training (**b**) and validation (**c**) cohort. Calibration curves of radiomics nomogram in the training (**d**) and validation (**e**) cohort. ALT, alanine transaminase; APRI, aspartate transaminase-to-platelet ratio index; AST, aspartate aminotransferase; FIB-4, fibrosis-4; GLOB, globulin; INR, international normalized ratio; ROC, receiver operating characteristic

As is shown in Fig. 4a, a radiomics-based nomogram integrated the radiomics signature, ALT (0, ≤25.9 U/L; 1, > 25.9 U/L), AST (0, ≤32.6 U/L; 1, > 32.5 U/L), GLOB (0, ≤33.9 g/L; 1, > 33.9 g/L) and INR (0, ≤1.10; 1, > 1.10). The formula of the radiomics model was: Y = 0.734 × ALT + 0.468 × AST + 1.385 × GLOB + 2.372 × Radiomics signature – 0.454.

ROC analyses comparing the discrimination ability of the radiomics-based nomogram to those of the CT-reported cirrhosis alone, APRI and FIB-4 are given in Fig. 4b, c. As summarized in Table 3, in the training cohort, the radiomics-based model had the best discriminatory ability with an AUC value of 0.915 (95% CI: 0.869, 0.961), which was significantly higher than that of the CT-reported cirrhosis alone (AUC: 0.752; 95% CI: 0.683, 0.821; *P* < .001), APRI (AUC: 0.752; 95% CI: 0.683, 0.821; *P* < .001) and FIB-4 (AUC: 0.664; 95% CI: 0.575, 0.753; *P* < .001). In the validation cohort, the radiomics nomogram also yielded the highest AUC of 0.872 (95% CI: 0.814, 0.930) compared with the CT-reported cirrhosis alone (AUC: 0.755; 95% CI: 0.683, 0.827; *P* = .006), APRI (AUC: 0.731; 95% CI: 0.649, 0.814; *P* = .003) and FIB-4 (AUC: 0.688; 95% CI: 0.601, 0.775; *P* < .001). The optimal cutoff value of 0.014 for the radiomics nomogram was determined at the point of the maximum Youden index from the entire cohort. The nomogram achieved the overall correctly classified rate of 82.0%, with a sensitivity of 77.5%, a specificity of 85.1%, positive predictive value of 78.2% and negative predictive value of 84.6%, respectively.

**Table 3:** Diagnostic Performances of All Methods for Predicting Liver Cirrhosis in the training and validation cohort

	Training (n = 144)	Validation (n = 150)	Training vs. Validation
**Methods**	**AUROC (95%CI)**	**AUROC (95%CI)**	**Delong test**
**Radiomics nomogram**	0.915 (0.869, 0.961)	0.872 (0.814, 0.930)	*P* = .257
**CT-reported cirrhosis status**	0.752 (0.683, 0.821)	0.755 (0.683, 0.827)	*P* = .961
**APRI**	0.725 (0.642, 0.809)	0.731 (0.649, 0.814)	*P* = .921
**FIB-4**	0.664 (0.575, 0.753)	0.688 (0.601, 0.775)	*P* = .705
**Comparison of AUROC (Delong test)**			
**Radiomics nomogram vs. CT-reported cirrhosis status**	*P* < .001	*P* = .006	
**Radiomics nomogram vs. APRI**	*P* < .001	*P* = .003	
**Radiomics nomogram vs. FIB-4**	*P* < .001	*P* < .001	
**CT-reported cirrhosis status vs. APRI**	*P* = .594	*P* = .651	
**CT-reported cirrhosis status vs. FIB-4**	*P* = .073	*P* = .201	
**APRI vs. FIB-4**	*P* = .040	*P* = .040	

Note. ——Data in parentheses are the 95% confidence interval. *APRI* aspartate transaminase-to-platelet ratio index, *AUROC* area under the receiver operating characteristic, *FIB-4* fibrosis-4

The calibration curve of the nomogram demonstrated great agreement between predicted and actual cirrhosis in the training cohort (Fig. 4d). The Hosmer-Lemeshow test yielded a *p* value of 0.317, indicating no departure from the good fit. The favorable calibration of the nomogram was further confirmed in the validation cohort by the calibration curve (Fig. 4e) and the Hosmer-Lemeshow test (*P* = 0.534).

**Fig. 5:**
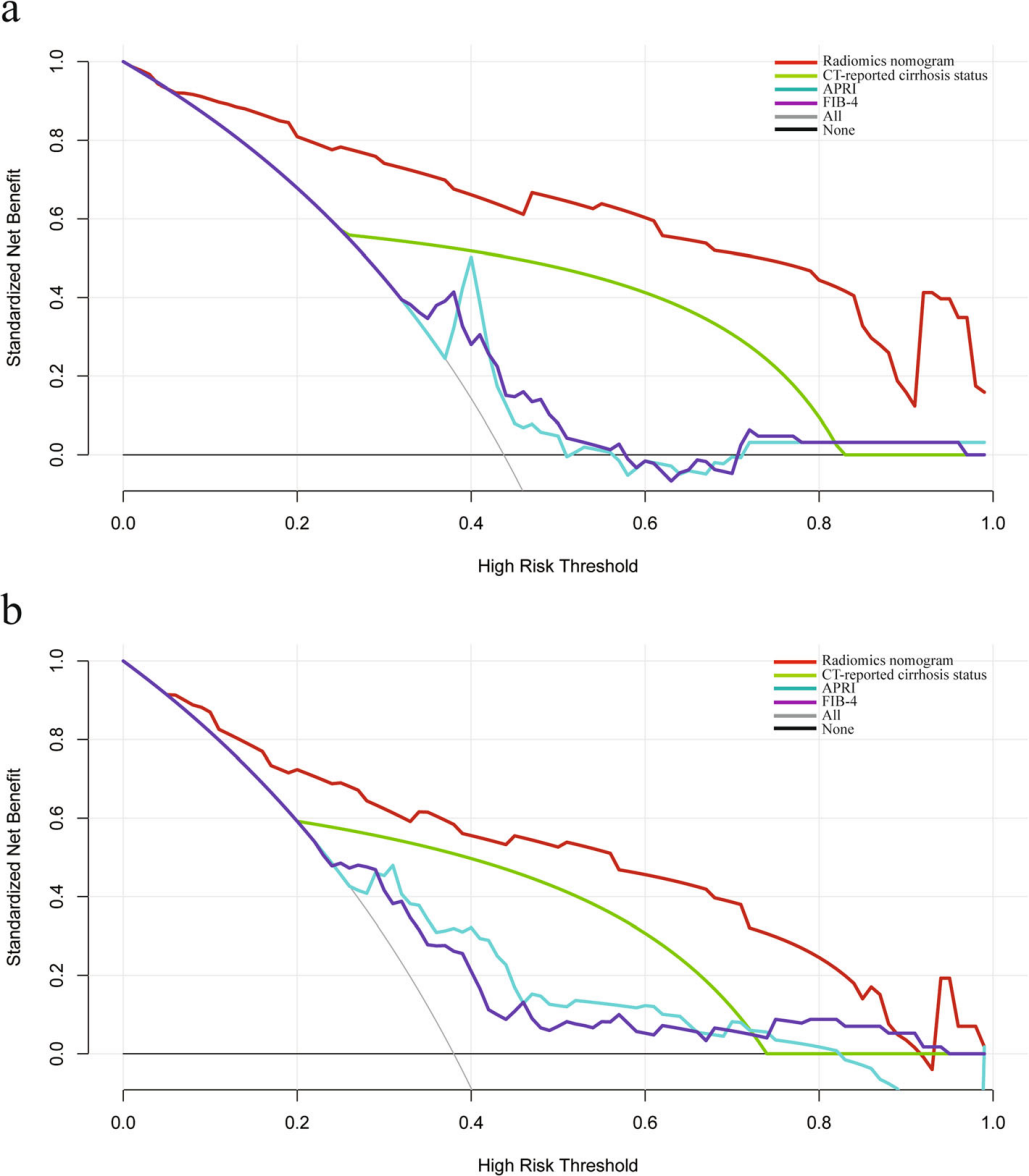
Decision curve analysis for each model in the training (**a**) and validation (**b**) dataset. The y-axis measures the net benefit. Across the threshold probability, the application of radiomics nomogram to predict cirrhosis status provides more benefit than treating all or none of the patients, CT-reported cirrhosis status alone, APRI and FIB-4. APRI, aspartate transaminase-to-platelet ratio index; FIB-4, fibrosis-4

DCA for the radiomics nomogram, CT-reported cirrhosis, APRI and FIB-4 is presented in Fig. 5. Across the range of reasonable threshold probabilities in both cohorts, DCA graphically demonstrated that the radiomics nomogram provided a larger net benefit to predict liver cirrhosis than the other three methods.

## Discussion

This study established and validated a radiomics-based model to predict liver cirrhosis in patients with HBV. The radiomics signature consisted of 25 stable radiomic features and had the great ability to identify patients as cirrhosis or non-cirrhosis cases. A user-friendly nomogram that integrates radiomics signature, ALT, AST, GLOB and INR achieved significantly better diagnostic performance and provided more clinical benefits compared with CT-reported cirrhosis alone, APRI and FIB-4.

Several less-invasive methods for staging liver fibrosis have been developed including serum indices and elastography. TE and MR elastography (MRE) are known to achieve great diagnostic performance for staging liver fibrosis [24, 25]. However, these well-performed modalities are not widely applied because of high prices. CT is frequently suggested for HBV carriers in China, but many patients only accept non-contrast CT examinations because of limited cost-effectiveness. Contrast-enhanced CT or MRI can provide more information than non-contrast CT, but we would like to develop noninvasive models for predicting cirrhosis on the basis of easily obtained data with relatively low cost. Not only the diagnostic performance but also the cost and applicability should be considered.

Previous studies detected the feasibility of computerized analysis of MRI and used radiomics to obtain great prediction results for liver fibrosis [17, 26]. CT is more readily available than MRI and Koichiro et al. investigated the predictive value for liver cirrhosis based on portal phase CT images using deep learning techniques, of which the AUC value was 0.73 (95% CI: 0.62, 0.84) [27]. In our study, we provided the first evidence of the feasibility of radiomics analysis of non-contrast CT and established a robust radiomics signature. Moreover, we included the independent serum predictors into the nomogram to provide more clinical benefits. ALT, AST, GLOB and INR have been reported in several cirrhosis-related studies. AST and ALT can help identify patients who requiring antiviral therapy prior to disease progression [28]. The AST to ALT ratio is frequently calculated for predicting fibrosis stage or clinical outcomes in chronic hepatitis [29, 30]. It was reported that globulin is positively associate with mortality in patients with cirrhosis [31]. Serum gamma-globulin ≥18 g/L is a significant predictor of disease progression, tumor development and death for cirrhotic patients [32]. INR is used to assess bleeding risk and prognosis in cirrhosis, and end-stage liver disease score that integrates INR has the ability to prioritize patients for liver transplantation [33].

The reproducibility of radiomic features was always worried by several researchers [34, 35]. Our study set up a reproducibility test, including intra- and interobserver ICC calculations with the minimum threshold of 0.80. Only 10.3% (85 of 828) radiomic features were included in the subsequent analysis in this study. The reason for this might be slightly different regions of interest delineated by two radiologists and relatively strict criteria of reproducibility test. Moreover, there would be a significant improvement in the clustering reproducibility of radiomic features, by selecting a smaller subset of more reproducible radiomic features [36].

This study focused on the radiomics-based prediction model based on non-contrast CT. Although CT is easily obtained and the model with great performance can become an alternative to elastography, it is more meaningful to detect the image biomarker of ultrasonography. Ultrasonography, as an annual physical examination item, is frequently the initial tool for liver tumor screening in patients with chronic hepatitis in the world. However, most radiomics techniques are only applied to three-dimensional images (CT, MRI, PET/CT) [22], and software that can systematically extract radiomic features from 2-D ultrasound images is rare. We are also developing the software that can extract radiomic features from 2-D images according to related articles [37]. In the future, we will also develop the model based on region of interest of ultrasound images for more clinicians.

Several limitations in this study should be noted. First, inherent selection biases cannot be avoided due to the retrospective nature of this study. Second, the radiomics-based nomogram was established and validated on the basis of data obtained from a single center. Multi-institutional studies are required for further validations. Third, this study only focused on the predictive value for liver cirrhosis, lacking significant & advanced fibrosis, leading to the limited clinical benefits. Two reasons for this: only cirrhosis can be evaluated by radiologists due to image findings on non-contrast CT; the diagnosis of cirrhosis remains more clinically significant compared with other fibrosis stage. Finally, the correlations of radiomic features with genomic patterns were not investigated.

In conclusion, we proposed a noninvasive and user-friendly radiomics-based model that integrates the radiomics signature based on non-contrast CT scans and independent serum indices to evaluate the liver cirrhosis status in patients with HBV. The radiomics model can help clinical decision making and potentially provide benefits for clinicians and selected patients.

## Supplementary information

**Additional file 1: Supplement materials and methods. Figure S1.** Non-contrast CT image of a 53-year-old man infected with HBV. The region of interest for the liver is delineated along the margin of the right hepatic lobe, at the level of the right portal vein, by excluding large hepatic vessels. The pink line indicates the region of interest. CT, computed tomography. HBV, hepatitis B virus.

**Additional file 2.**

## Data Availability

The data is not available because of patients’ privacy.

## Abbreviations

AASLD: American Association for the Study of Liver Diseases
ALP: Alkaline phosphatase
ALT: Alanine aminotransferase
Apo A1: Apolipoprotein A1
Apo B: Apolipoprotein B
APRI: Aspartate transaminase-to-platelet ratio index
AST: Aspartate aminotransferase
AUC: Area under the curve
AUROC: Area under the receiver operating characteristic curve
CHB: Chronic hepatitis B
CI: Confidence interval
CRP: C reactive protein
CT: Computed tomography
DCA: Decision curve analysis
EASL: European Association for the Study of Liver
FIB-4: Fibrosis-4
GGT: Gamma-glutamyl transpeptidase
GLOB: Globulin
Hb: Hemoglobin
HBsAg: Hepatitis B surface antigen
HBV: Hepatitis B virus
HCV: Hepatitis C virus
HDV: Hepatitis D virus
HDL-C: High density lipoprotein cholesterol
HIV: Human immunodeficiency virus
ICC: Intraclass correlation coefficient
INR: International normalized ratio
LAP: Leucine arylamidase
LASSO: The least absolute shrinkage and selection operator
LDH: Lactate dehydrogenase
LDL-C: Low density lipoprotein cholesterol
MRE: Magnetic resonance elastography
MRI: Magnetic resonance image
PLT: Platelet
PT: Prothrombin time
RBC: Red blood cell
R-cirrhosis: The radiomics model for predicting liver cirrhosis
ROI: Region of interest
SVM: Support vector machine
TB: Serum total bilirubin
TBA: Total bile acid
TC: Total cholesterol
TE: Transient elastography
ULN: The upper limit of normal
WBC: White blood cell
WHO: World health organization
