# Supplementary material for: A radiomics-based model on non-contrast CT for predicting cirrhosis: make the most of image data
Source: Biomark Res. 2020 Sep 17;8:47. doi: 10.1186/s40364-020-00219-y (PMC7499912; doi:10.1186/s40364-020-00219-y)
Supplement: Supplementary file 1 — Additional file 1: Supplement materials and methods. Figure S1. Non-contrast CT image of a 53-year-old man infected with HBV. The region of interest for the liver is delineated along the margin of the right hepatic lobe, at the level of the right portal vein, by excluding large hepatic vessels. The pink line indicates the region of interest. CT, computed tomography. HBV, hepatitis B virus. [file 40364_2020_219_MOESM1_ESM.docx]

**Supplement materials and methods**

1. **Details of two-step procedure for selection of clinical factors**

The two-step procedure includes spearman correlation analysis and subsequent forward conditional logistic multivariable analysis.

**1.1 Spearman correlation analysis**

Spearman correlation analysis is a statistical measure of the strength of a monotonic relationship between paired data. It is frequently used for ordered categorical data (e.g. non-cirrhosis, 0; cirrhosis, 1). In a sample, it is denoted by *r* and is by design constrained as -1 ≤ *r* ≤ 1. The closer *r* is to ±1 the stronger the monotonic relationship. Correlation is an effect size and the strength of the correlation is verbally described according to the following guide for the absolute value of *r*: 0.00-0.19, “very weak”; 0.20-0.39, “weak”; 0.40-0.59, “moderate”; 0.60-0.79, “strong”; 0.80-1.0, “very strong”.

*r^2^* is the square of *r*, and it is named as the coefficient of determination. The result of *r^2^* is preferred to be expressed in correlation analysis (such as the scientific style guide of *Radiology* [1]) because the coefficient of determination measures the fraction of the total variation in the dependent variable that is explained by the independent variable. In addition, a significance test should be performed to decide whether based upon this sample there is any or no evidence to suggest that linear correlation is present in the population. *P* < .05 was considered statistically significant.

- 1. **Forward conditional logistic multivariable analysis**

Factors that were confirmed related to cirrhosis status using spearman correlation analysis (*P* < .05), would be subsequently included into the forward conditional logistic multivariable analysis. Spearman correlation analysis can only determine the direct relationship between factors and cirrhosis status, but it cannot check for the collinearity of factors. Confounding occurs when the apparent association between a factor and an outcome is affected by the relationship of a third variable to the factor and to the outcome; the third variable is named as confounder [2].

Multivariable analysis is a statistical tool for determining the unique contributions of various factors to a single event or outcome [3]. In this study, it can allow us to determine the independent contribution of each of included factors to the development of liver cirrhosis. Logistic regression, as one of the three types of multivariable analysis, is used with dichotomous outcomes (binary variable). The cirrhosis status is in form of binary variable, and thus logistic multivariable analysis was conducted. The input and output *P* value for the forward conditional logistic regression is .05 and .10, respectively.

1. **Details of development and validation of a radiomics-based model**

Multivariable logistic regression analysis was used to develop a model for predicting liver cirrhosis in the training cohort. To provide a more understandable established models, a nomogram was subsequently constructed by using the selected clinical factors and radiomics signature.

Diagnostic performance was evaluated in terms of discrimination ability, calibrations and clinical usefulness. The discrimination ability of the established model was quantified by the receiver operating characteristic (ROC) curve and area under the curve (AUC) value [4]. Delong test was used to compare AUC values of prediction methods [5]. Calibration curves were plotted via bootstrapping with 1000 resamples to asses the calibration of the radiomics-based model, accompanied by the Hosmer-Lemeshow goodness-of fit test. Clinical usefulness was evaluated by decision curve analysis (DCA), which can calculate the net benefit provided by the use of all prediction methods at different threshold probabilities [6]. The performance of the radiomics-based model was then internally validated in an independent validation cohort by using the formula derived from the training cohort.

1. **Details of support vector machine**

The type of support vector machine (SVM) is “c-classification”, of which the kernel function is radial basis. The total number of support vectors is 133: 62 for non-cirrhosis and 71 for cirrhosis.


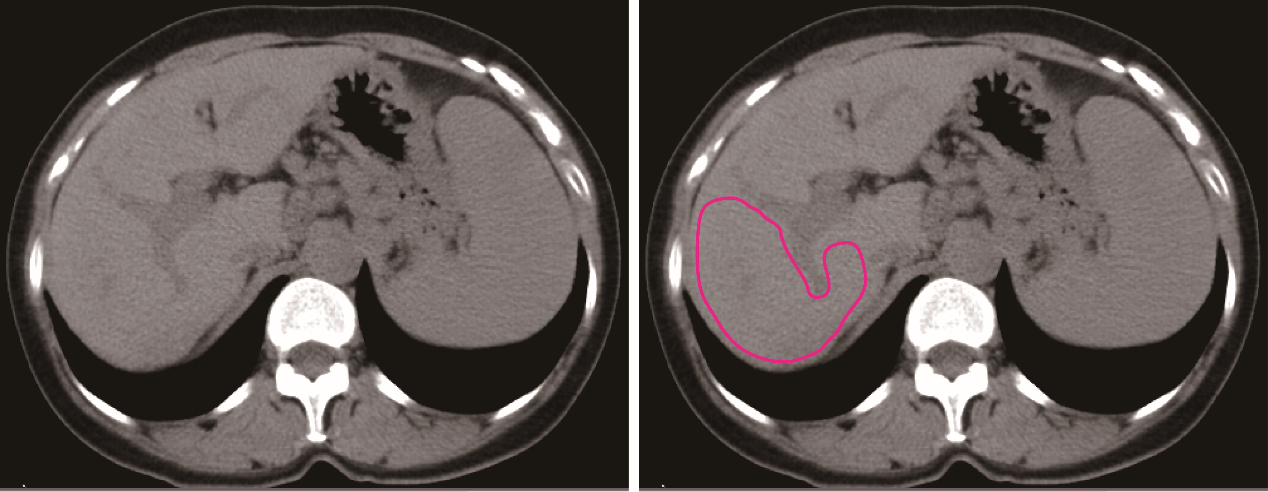


**Figure S1:** Non-contrast CT image of a 53-year-old man infected with HBV. The region of interest for the liver is delineated along the margin of the right hepatic lobe, at the level of the right portal vein, by excluding large hepatic vessels. The pink line indicates the region of interest. CT, computed tomography. HBV, hepatitis B virus.

**References**

[1] Radiological Society of North America. (2020). Scientific Style Guide. Retrieved from: <https://pubs.rsna.org/page/radiology/author-instructions>.

[2] Katz MH. Multivariable Analysis: A Practical Guide for Clinicians. Cambridge: Cambridge Univ Pr; 1999.

[3] Katz MH. Multivariable analysis: a primer for readers of medical research. *Ann Intern Med*. 2003;138(8):644-650.

[4] Hanley JA, McNeil BJ. The meaning and use of the area under a receiver operating characteristic (ROC) curve. Radiology 1982;143(1):29–36.

[5] DeLong ER, DeLong DM, Clarke-Pearson DL. Comparing the areas under two or more correlated receiver operating characteristic curves: a nonparametric approach. Biometrics 1988;44(3):837–845.

[6] Steyerberg EW, Vickers AJ. Decision curve analysis: a discussion. Med Decis Making 2008;28(1):146–149.
