# Supplementary material for: A radiomics-based model on non-contrast CT for predicting cirrhosis: make the most of image data
Source: Biomark Res. 2020 Sep 17;8:47. doi: 10.1186/s40364-020-00219-y (PMC7499912; doi:10.1186/s40364-020-00219-y)
Supplement: Supplementary file 2 — Additional file 2. [file 40364_2020_219_MOESM2_ESM.docx]

**Explanation of Three Corresponding Authors**

Beicheng Sun, Department of Hepatobiliary Surgery, The Affiliated Drum Tower Hospital of Nanjing University Medical School, 321 Zhongshan Road, Nanjing 210008, Jiangsu Province. Email: [sunbc@nju.edu.cn](mailto:sunbc@nju.edu.cn); Telephone number: +862583105987; Fax number: +862583105987;

Jian He, Department of Radiology, The Affiliated Drum Tower Hospital of Nanjing University Medical School, 321 Zhongshan Road, Nanjing 210008, Jiangsu Province. Email: [hjxueren@163.com](mailto:hjxueren@163.com); Telephone number: +862583105987; Fax number: +862583105987;

Jun Chen, Department of Pathology, The Affiliated Drum Tower Hospital of Nanjing University Medical School, 321 Zhongshan Road, Nanjing 210008, Jiangsu Province. Email: [chenjun@njglyy.com](mailto:chenjun@njglyy.com). Telephone number: +862583105987; Fax number: +862583105987;

These three corresponding authors belong to the same medical institution (The Affiliated Drum Tower Hospital of Nanjing University Medical School), but they are from different departments (Beicheng Sun: Department of Hepatobiliary Surgery; Jian He: Department of Radiology; Jun Chen: Department of Pathology).

Actually, we used clinical data (basic characteristics and serum examination results), image data (radiomics features) and pathologic data (histologic results of liver biopsy) in this study. These three corresponding authors are all involved in the research design and manuscript editing. Beicheng Sun, as a leader of Department of Hepatobiliary Surgery, provided the clinical data and supervised the work of patients’ enrollment, data collection and data analysis. Jian He (senior radiologist) organized his group to collect image data, delineate the region of interest and extract radiomic features. Jun Chen (senior pathologist) organized his group to collect pathological data and reassess pathological scores of biopsy samples.

All authors approved this arrangement.
